# Supplementary material for: Novel nucleoside analogs exhibit potent intracellular and in vivo activities against Mycobacterium avium
Source: Microbiol Spectr. 2026 Jan 23;14(3):e02160-25. doi: 10.1128/spectrum.02160-25 (PMC12955403; doi:10.1128/spectrum.02160-25)
Supplement: Supplemental figures and tables — Figures S1 to S6 and Tables S1 and S2. [file spectrum.02160-25-s0001.docx]

**
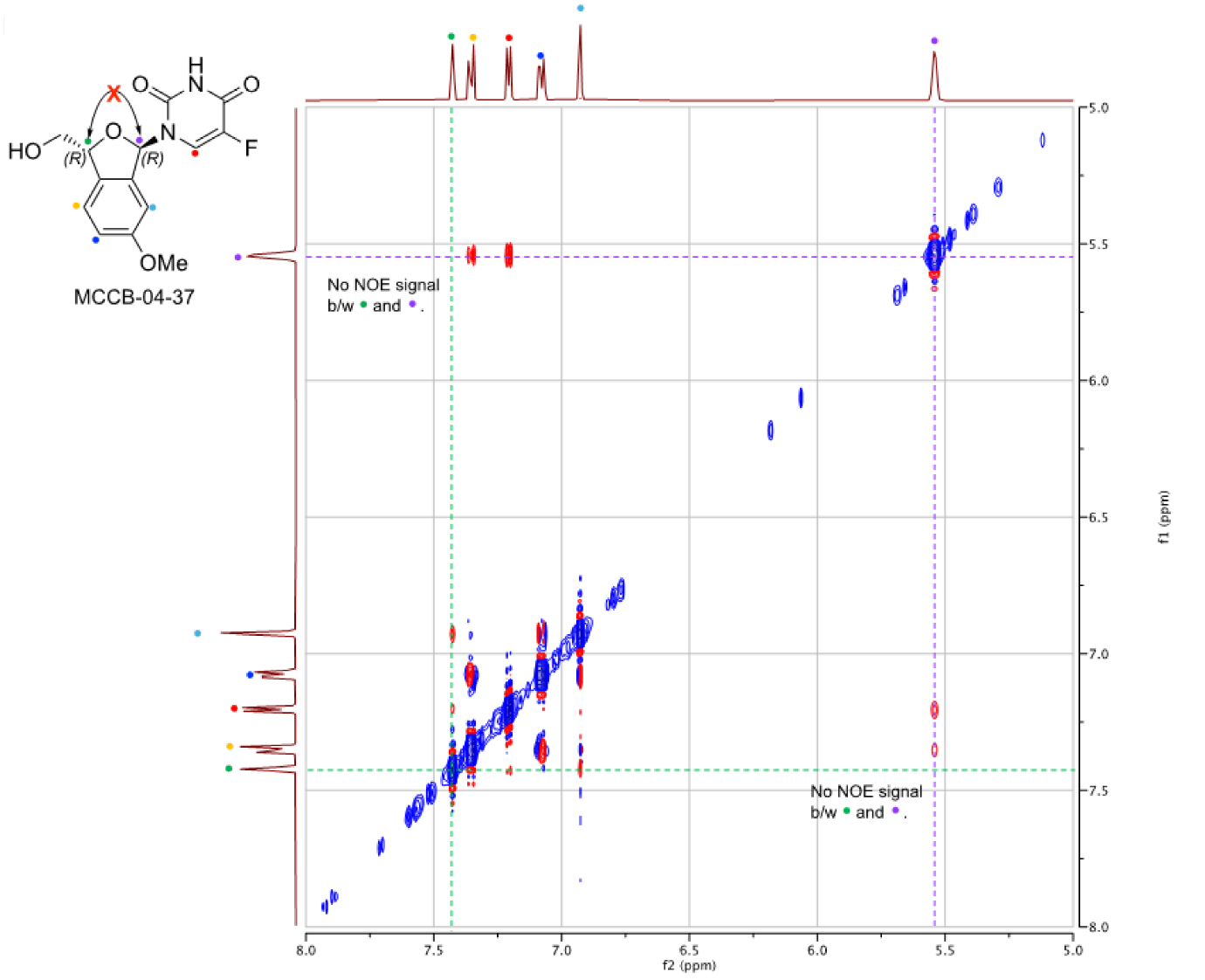
Figure S1. 2D NOESY spectrum of compound MCCB-04-037 (4b).** No NOE correlation between the protons at the two stereogenic centers was observed, consistent with a trans configuration. Blue and red cross-peaks indicate positive and negative NOE correlations, respectively.


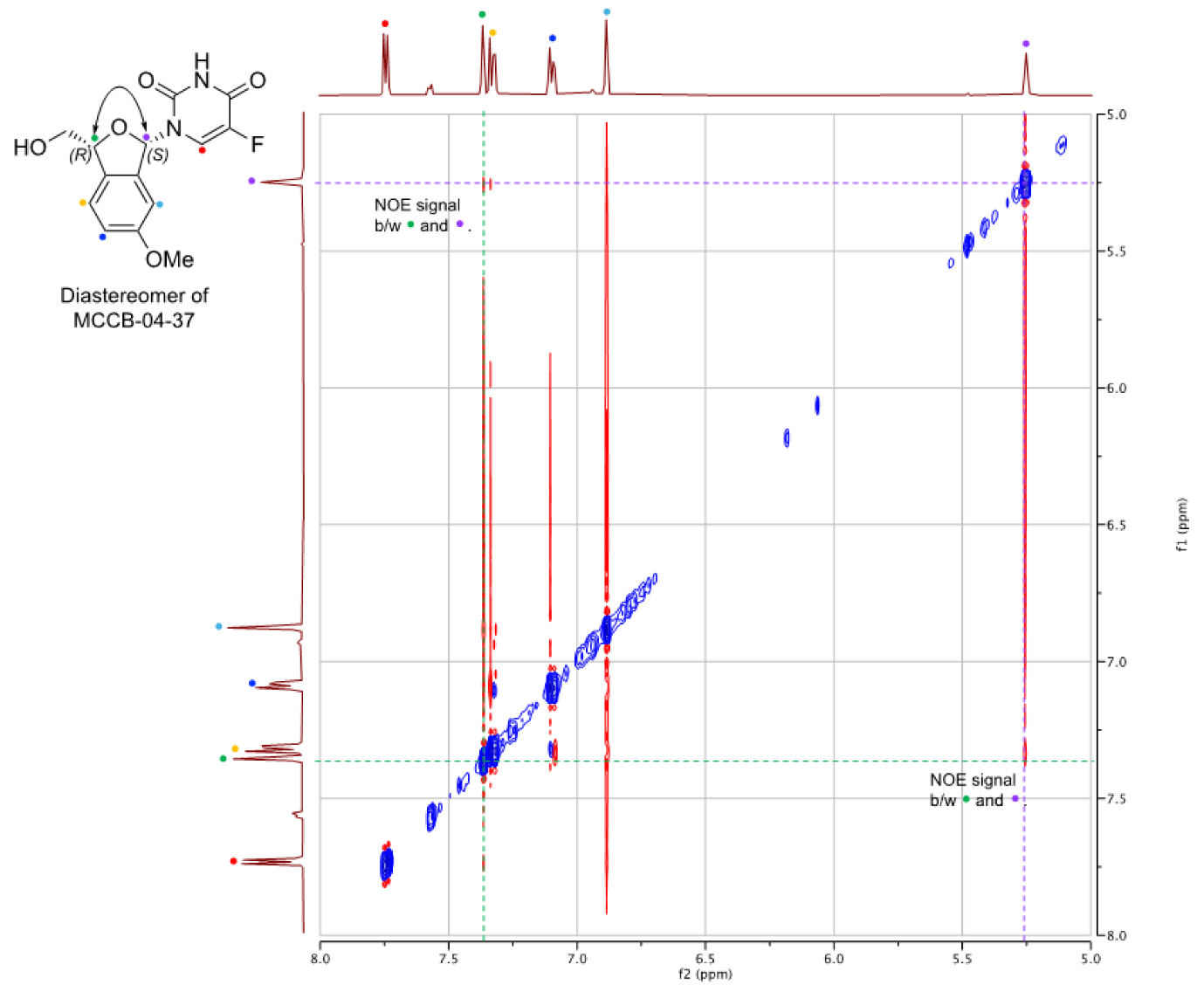
**Figure S2. 2D NOESY spectrum of the diastereomer of MCCB-04-037 (4b′).** A clear NOE correlation between the protons at the two stereogenic centers was observed, consistent with a *cis* configuration. Blue and red cross-peaks indicate positive and negative NOE correlations, respectively.


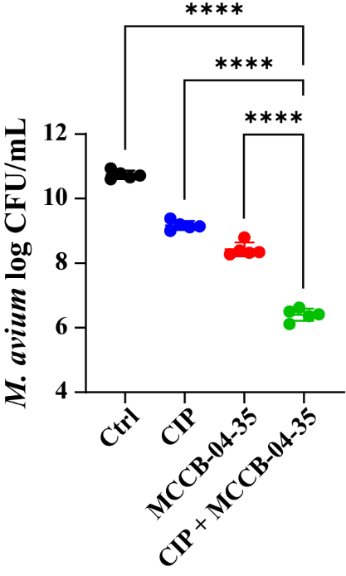

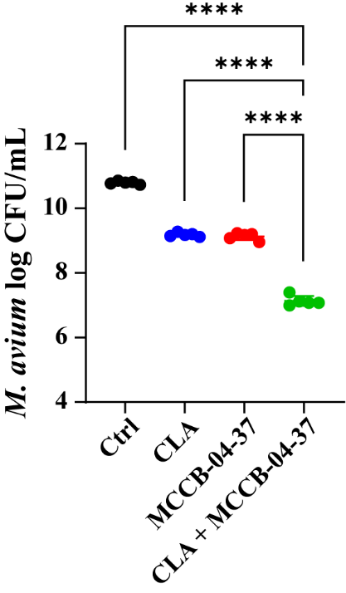


(A)

(B)

**Figure S3. CFU-based assay for evaluating synergistic effects of MCCB-04-35 and MCCB-04-37 with ciprofloxacin or clarithromycin against *Mycobacterium avium*.** (A) *M. avium* was treated with ciprofloxacin (CIP, 4 µg/mL), MCCB-04-35 (50 µg/mL), or their combination (CIP (1 µg/mL) + MCCB-04-35 (12.5 µg/mL)) for 72 h. (B) *M. avium* was treated with clarithromycin (CLA, 0.25 µg/mL), MCCB-04-37 (50 µg/mL), or their combination (CLA (0.06 µg/mL) + MCCB-04-37 (12.5 µg/mL)) for 72 h. Bacterial viability was evaluated by CFU count on 7H10 agar. Data represent mean ± SD from five independent replicates. Statistical analysis was performed using one-way ANOVA followed by Dunnett’s multiple-comparisons test.

(A)

(B)

(C)

**Figure S4. Antibacterial and anti-inflammatory effects of MCCB-04-35 and MCCB-04-37 at 1 week post-treatment in a chronic *Mycobacterium avium* infection model.** (A) Bacterial burden (log₁₀ CFU/mL) in lung tissue at 2 weeks post-infection. Mice were infected with *M. avium*-loaded beads and treated with MCCB-04-35 or MCCB-04-37. Lung homogenates were collected to determine *M. avium* colony counts. (B–C) Cytokine concentrations in bronchoalveolar lavage fluid (BALF). Tumor necrosis factor-α (TNF-α, B) and interferon-γ (IFN-γ, C) levels were measured to evaluate the host inflammatory response. Treatment with MCCB-04-35 and MCCB-04-37 significantly reduced bacterial load and cytokine expression. Data represent individual mice (*n* = 5). Statistical significance is indicated as follows: **p* < 0.05, ***p* < 0.01, ****p* < 0.001.

(A)

(B)

(C)

**Figure S5.** **Antibacterial and anti-inflammatory effects of MCCB-04-35 and MCCB-04-37 at 2 week post-treatment in a chronic *Mycobacterium avium* infection model.** (A) Bacterial burden (log₁₀ CFU/mL) in lung tissue at 3 weeks post-infection. Mice were infected with *M. avium*-loaded beads and treated with MCCB-04-35 or MCCB-04-37. Lung homogenates were collected to determine *M. avium* colony counts. (B–C) Cytokine concentrations in bronchoalveolar lavage fluid (BALF). Tumor necrosis factor-α (TNF-α, B) and interferon-γ (IFN-γ, C) levels were measured to evaluate the host inflammatory response. Treatment with MCCB-04-35 and MCCB-04-37 significantly reduced bacterial load and cytokine expression. Data represent individual mice (*n* = 5). Statistical significance is indicated as follows: **p* < 0.05, ***p* < 0.01, ****p* < 0.001.

**
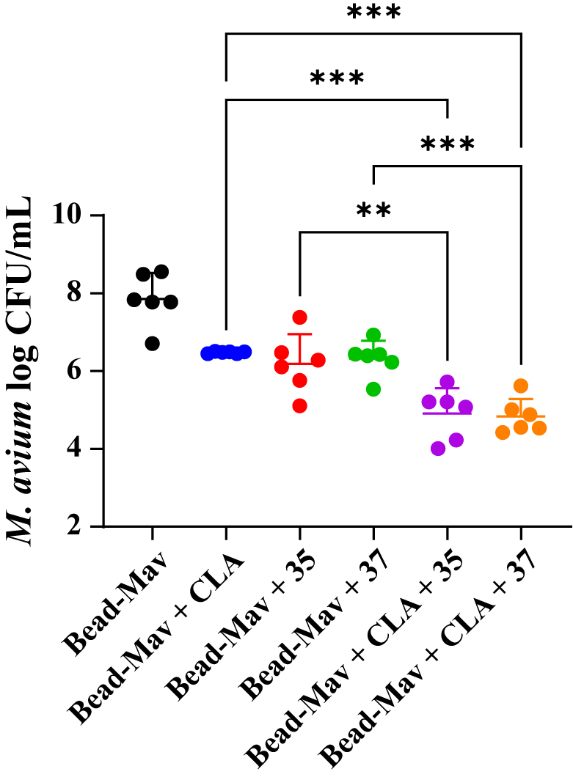
**

**Figure S6. Combination treatment of MCCB-04-35 or MCCB-04-37 with clarithromycin in a chronic *Mycobacterium avium* infection model.** Mice were infected *via* tracheal intubation with *M. avium*–loaded agar beads (Bead-Mav) and treated intraperitoneally for 3 weeks with clarithromycin (25 mg/kg), MCCB-04-35 (25 mg/kg), MCCB-04-37 (25 mg/kg), or their combinations. Pulmonary bacterial burdens were assessed by plating lung homogenates on Middlebrook 7H10 agar, and colony-forming units (CFU) were expressed as log₁₀ CFU/mL. Each pair of dots represents data from one mouse. Bars indicate mean ± SD. Statistical significance was analyzed by one-way ANOVA with Dunnett’s post-hoc test (***p* < 0.01, ****p* < 0.001).

**Supplementary Table 1. Minimal inhibitory concentrations (MICs) of MCCB-04-35 and MCCB-04-37 against various nontuberculosis mycobacterial strains.**

| **Code** | **ATCC 19977** | **ATCC 104** | **00136-61038** | **00136-41015** | **00136-52005** | **00136-60005** | **00136-43008** |
| --- | --- | --- | --- | --- | --- | --- | --- |
| **Strain** | ***M. abscessus*** | ***M. avium*** | ***M. abscessus*** | ***M. avium*** | ***M. bolletii*** | ***M. fortuitum*** | ***M. intracellulare*** |
| **MIC (ug/ml)** | **standard** | **standard** | **clinical** | **clinical** | **clinical** | **clinical** | **clinical** |
| **Clarithromycin** | 0.5 | 0.25 | ≥64 | 16 | ≥64 | ≥64 | 0.25 |
| **Amikacin** | 8 | 0.5 | 8 | 32 | 32 | 8 | 32 |
| **Moxifloxacin** | 0.25 | 8 | ≥8 | ≥8 | ≥8 | 0.5 | ≥8 |
| **Linezolid** | 8 | 4 | 2 | ≥64 | ≥64 | ≥64 | ≥64 |
| **Ciprofloxacin** | 4 | 4 | ≥8 | ≥8 | ≥8 | ≥8 | ≥8 |
| **MCCB-04-35** | ≥320 | 20 | ≥320 | ≥320 | ≥320 | ≥320 | 5 |
| **MCCB-04-37** | 10 | 40 | 10 | ≥320 | ≥320 | ≥320 | 20 |

The minimum inhibitory concentrations (MICs, µg/mL) of MCCB-04-35 and MCCB-04-37 were determined against standard and clinical isolates of nontuberculous mycobacteria (NTM), including *M. abscessus, M. avium, M. bolletii, M. fortuitum,* and *M. intracellulare*, using the broth microdilution method following CLSI guidelines (M24-A2). Clarithromycin, amikacin, moxifloxacin, linezolid, and ciprofloxacin were used as reference antibiotics. MIC was defined as the lowest concentration that completely inhibited visible bacterial growth after 7 days of incubation at 37 °C. Data represent the results from three independent experiments.

**Supplementary Table 2. Differential expression of genes associated with representative GO terms and KEGG pathways upon MCCB-04-35 and MCCB-04-37 treatment**

| locus_tag | pathway | GO | MCCB-04-35 | | MCCB-04-37 | |
| --- | --- | --- | --- | --- | --- | --- |
|  |  |  | Fold Change | p-value | Fold Change | p-value |
| MAV_RS00805 | Nitrogen metabolism | glutamate synthase (NADPH) activity;L-glutamate biosynthetic process | -3.569 | 4.48E-05 | -4.914 | 3.74E-09 |
| MAV_RS00810 | Nitrogen metabolism | glutamate synthase (NADPH) activity;L-glutamate biosynthetic process | -6.167 | 6.62E-09 | -6.627 | 2.07E-09 |
| MAV_RS07285 | Oxidative phosphorylation | proton-transporting ATPase activity;proton-transporting ATP synthase complex | -3.169 | 4.60E-04 | -1.592 | 2.24E-02 |
| MAV_RS07290 |  | proton-transporting ATP synthase complex | -2.486 | 6.63E-02 | -2.710 | 4.65E-02 |
| MAV_RS07295 | Oxidative phosphorylation | proton-transporting ATPase activity;proton-transporting ATP synthase complex | -1.303 | 9.14E-03 | -3.939 | 1.59E-03 |
| MAV_RS14965 | Nucleotide excision repair |  | 2.806 | 1.04E-07 | 2.260 | 7.30E-05 |
| MAV_RS14995 | Nucleotide excision repair |  | 1.858 | 5.86E-08 | 1.717 | 1.05E-07 |
| MAV_RS16055 | Nucleotide excision repair |  | 5.680 | 2.62E-02 | 5.077 | 5.29E-02 |
| MAV_RS07230 | RNA degradation |  | 0.776 | 4.79E-02 | 1.330 | 5.82E-03 |
| MAV_RS08265 | RNA degradation |  | 2.345 | 1.45E-08 | 1.586 | 5.79E-04 |
| MAV_RS23105 | RNA degradation |  | 1.974 | 2.12E-03 | 2.098 | 3.41E-03 |
| MAV_RS06245 | Nitrogen metabolism |  | -3.341 | 1.30E-02 | -6.864 | 1.83E-12 |
| MAV_RS23575 | Nitrogen metabolism |  | -11.450 | 1.78E-18 | -11.553 | 1.06E-18 |
| MAV_RS18730 | Oxidative phosphorylation |  | -4.783 | 1.05E-04 | -5.452 | 3.24E-06 |
| MAV_RS19330 | Oxidative phosphorylation |  | -8.189 | 1.44E-04 | -7.339 | 1.82E-04 |
| MAV_RS19385 | Oxidative phosphorylation |  | -8.647 | 2.72E-03 | -8.712 | 4.78E-03 |
| MAV_RS22930 | Oxidative phosphorylation |  | -7.312 | 4.01E-02 |  |  |

RNA was extracted from *Mycobacterium avium* cultures treated with each compound and subjected to bulk RNA sequencing. Reads were quality-trimmed using Trimmomatic, aligned to the MAH 104 reference genome with STAR, and quantified using feature counts. Differential expression analysis was performed using DESeq2, and pathway enrichment was conducted via FUNAGE-Pro. Genes listed in this table are annotated to pathways and GO terms enriched in Fig. 4C–F, with adjusted *p*-values < 0.05.
