# Supplementary material for: Novel nucleoside analogs exhibit potent intracellular and in vivo activities against Mycobacterium avium
Source: Microbiol Spectr. 2026 Jan 23;14(3):e02160-25. doi: 10.1128/spectrum.02160-25 (PMC12955403; doi:10.1128/spectrum.02160-25)
Supplement: Supplemental material — s and Methods. [file spectrum.02160-25-s0002.docx]

**Synthetic Procedures**

**Materials and General Methods**

Unless otherwise noted, all reactions were conducted in an oven-dried glassware under inert atmospheric conditions with anhydrous solvents. All reactions were monitored by analytical thin-layer chromatography (TLC) using a precoated silica aluminum plate with F254 indicators, and the product profiles were visualized by UV irradiation (254 nm) and staining with phosphomolybdic acid, ninhydrin, and/or potassium permanganate solution. The solvents including dichloromethane, toluene, and ethyl ether were dried by being passed through activated alumina column. Anhydrous other solvents were purchased from Sigma-Aldrich (Missouri, USA) or Alfa-Aesar (Massachusetts, USA). All other chemicals were purchased from Sigma-Aldrich, Alfa Aesar, TCI Chemicals (Japan), AK scientific (California, USA), or Daejung Chemicals & Metals (Republic of Korea), and they were used as received unless otherwise noted.

The ^1^H NMR, ^13^C NMR and 2D NOESY data were recorded using Bruker Advance 500 (Bruker, Munich, Germany). Chemical shifts were reported in parts per million (ppm) relative to chloroform (^1^H: 7.26 ppm, ^13^C: 77.16 ppm), methanol (^1^H: 3.31 ppm, ^13^C: 49.00 ppm), dimethyl sulfoxide (^1^H: 2.50 ppm, ^13^C: 39.52 ppm), or tetramethylsilane (TMS, 0.00 ppm), and the coupling constants were reported in Herz (Hz). High-resolution mass spectra were collected using Bruker Compact QTOF (Bruker, Munich, Germany), where the electrospray ionization method was employed for ionization.

Both analytical and preparative high performance liquid chromatography (HPLC) experiments were conducted using a Thermo Ultimate 3000 equipped with a diode array detector (commonly, the absorbance at 215 nm, 254 nm, and 280 nm was monitored). For analysis and purification, Acclaim 5 µm C18 120 Å column (Thermo Fisher scientific, 150 × 4.6 mm, Cat# 05941) and Luna 5 μm C18(2) 100 Å column (Phenomenex, 250 × 10 mm, Cat# 00G-4252-N0) were used, respectively.

**General procedure for compound 2a/2b**

To a solution of compound **1a** (1.0 eq.) in toluene (0.3 M) was added 1,3-propandiol (1.0 eq.) and *p*-toluenesulfonic acid (*p*-TsOH, 0.012 eq.) at room temperature. The reaction was stirred for 12 h at 120 ºC (Dean–Stark). After reaction, reaction mixtures were quenched by triethylamine (Et_3_N, 5.0 eq.). Then, the solvent was evaporated *in vacuo*. The resulting residue was diluted with diethylether (Et_2_O), washed with brine and dried over anhydrous magnesium sulfate. Then, vinyl benzaldehyde intermediate was used without further purification. To a solution of intermediate (1.0 eq.) in toluene (0.2 M) was added potassium vinyltrifluoroborate (1.2 eq.), Et_3_N (3.0 eq.), Pd_2_(dba)_3_ (0.005 eq.) and RuPhos (0.01 eq.) at room temperature. The reaction was stirred overnight at 115 ºC with reflux set up. After reaction, the resulting residue was diluted with ethyl acetate (EtOAc), washed with brine, and dried over anhydrous magnesium sulfate. After concentrated under pressure, the crude mixture was purified by flash column chromatography (SiO_2_, Hexane: EtOAc = 9:1) to afford the desired product compound **2a/2b** (59–69% yield, 2 steps).

**2-(5-methyl-2-vinylphenyl)-1,3-dioxane (2a)**

Compound **2a** (3.45 mmol, 69% yield, 2 steps) was synthesized from **1a** (5.00 mmol) following the described above general procedure. ^1^H NMR (500 MHz, CDCl_3_) δ 7.44 – 7.42 (m, 1H), 7.40 (d, *J* = 7.9 Hz, 1H), 7.13 – 7.11 (m, 1H), 7.08 (dd, *J* = 17.7, 11.2 Hz, 1H), 5.66 (s, 1H), 5.62 (dd, *J* = 17.4, 1.4 Hz, 1H), 5.28 (dd, *J* = 11.0, 1.4 Hz, 1H), 4.30 – 4.25 (m, 2H), 3.99 (td, *J* = 12.4, 2.5 Hz, 2H), 2.34 (s, 3H), 2.32 – 2.21 (m, 1H), 1.48 – 1.43 (m, 1H). ^13^C NMR (125 MHz, CDCl_3_) δ 137.72, 135.20, 134.03, 133.35, 129.76, 126.79, 126.03, 115.49, 100.21, 67.67, 25.90, 21.33. HR-MS (ESI-TOF) *m/z* for [C_13_H_16_NaO_2_]^+^ ([M+Na]^+^): calculated 227.1048, measured 227.1037.

**2-(5-methoxy-2-vinylphenyl)-1,3-dioxane (2b)**

Compound **2b** (2.95 mmol, 59% yield, 2 steps) was synthesized from **1b** (5.00 mmol) following the described above general procedure. ^1^H NMR (500 MHz, CDCl_3_) δ 7.44 (d, *J* = 8.6 Hz, 1H), 7.17 (d, *J* = 2.8 Hz, 1H), 7.02 (dd, *J* = 17.4, 11.0 Hz, 1H), 6.86 (dd, *J* = 8.6, 2.8 Hz, 1H), 5.65 (s, 1H), 5.56 (dd, *J* = 17.4, 1.3 Hz, 1H), 5.23 (dd, *J* = 11.0, 1.3 Hz, 1H), 4.31 – 4.25 (m, 2H), 3.99 (td, *J* = 12.4, 2.4 Hz, 2H), 3.83 (s, 3H), 2.26 (qt, *J* = 12.6, 5.0 Hz, 1H), 1.48 – 1.41 (m, 1H). ^13^C NMR (125 MHz, CDCl_3_) δ 159.45, 136.73, 133.59, 128.86, 127.36, 115.56, 114.45, 110.75, 99.84, 67.63, 55.50, 25.85. HR-MS (ESI-TOF) *m/z* for [C_13_H_16_NaO_3_]^+^ ([M+Na]^+^): calculated 243.0997, measured 243.0975.

**General procedure for compound 3a/3b**

AD-mix β (1.8 eq.) was dissolved clearly in *t*-butylalcohol : H_2_O = 1:1 solution (0.1 M) at room temperature. Then the solution cools down to –10 ºC and was add compound **2a/2b** (1.0 eq.). After addition of compound **2a/2b** the reaction mixture was stirred for 1 h at 0 ºC. Then the reaction was quenched by sodium sulfite (Na_2_SO_3_, 12 eq.) was added and stirred 0.5 h for quenching. Then mixture was diluted with dichloromethane (CH_2_Cl­_2_) and washed with H_2_O. And dried over anhydrous magnesium sulfate. After concentrating under pressure, the residue purified by flash column chromatography (SiO_2_, Hexane: EtOAc = 2:1). Purified diol intermediate (1.0 eq.) was dissolved in toluene (0.2 M) and then add triethylamine (10 eq.) at –20 ºC. Then pivaloyl chloride (1.2 eq.) was add dropwise and stirred overnight. After reaction, mixture was quenched by ice-water and stirred for 0.5 h at room temperature. Then extract with CH_2_Cl_2_ and dried over anhydrous magnesium sulfate. After concentrated under pressure, the residue purified by flash column chromatography (SiO_2_, Hexane: EtOAc = 4:1) to afford compound **3a/3b** (52–58% yield, 2 steps).

**(*R*)-2-(2-(1,3-dioxan-2-yl)-4-methylphenyl)-2-hydroxyethyl pivalate (3a)**

Compound **3a** (1.42 mmol, 58% yield, 2 steps) was synthesized from **2a** (2.45 mmol) following the described above general procedure. ^1^H NMR (500 MHz, CDCl_3_) δ 7.45 – 7.41 (m, 1H), 7.40 (d, *J* = 1.3 Hz, 1H), 7.18 (dd, *J* = 7.9, 1.3 Hz, 1H), 5.70 (s, 1H), 5.36 (dd, *J* = 7.9, 3.4 Hz, 1H), 4.42 (dd, *J* = 11.4, 3.7 Hz, 1H), 4.30 – 4.24 (m, 2H), 4.20 (dd, *J* = 11.4, 8.1 Hz, 1H), 4.06 – 3.97 (m, 2H), 2.75 (s, 1H), 2.34 (s, 3H), 2.25 (qt, *J* = 12.5, 5.0 Hz, 1H), 1.47 (ddd, *J* = 12.4, 2.5, 1.3 Hz, 1H), 1.22 (s, 9H). ^13^C NMR (125 MHz, CDCl_3_) δ 178.82, 137.96, 135.63, 135.25, 129.99, 127.50, 126.97, 100.80, 68.79, 68.67, 67.66, 67.59, 38.98, 27.36, 25.81, 21.23. HR-MS (ESI-TOF) *m/z* for [C_18_H_26_NaO_5_]^+^ ([M+Na]^+^): calculated 345.1678, measured 345.1681.

**(*R*)-2-(2-(1,3-dioxan-2-yl)-4-methoxyphenyl)-2-hydroxyethyl pivalate (3b)**

Compound **3b** (1.18 mmol, 52% yield, 2 steps) was synthesized from **2b** (2.27 mmol) following the described above general procedure. ^1^H NMR (500 MHz, CDCl_3_) δ 7.45 (d, *J* = 8.6 Hz, 1H), 7.14 (d, *J* = 2.8 Hz, 1H), 6.90 (dd, *J* = 8.6, 2.8 Hz, 1H), 5.69 (s, 1H), 5.31 (dd, *J* = 7.9, 3.6 Hz, 1H), 4.40 (dd, *J* = 11.4, 3.8 Hz, 1H), 4.27 (dt, *J* = 10.9, 4.5 Hz, 2H), 4.19 (dd, *J* = 11.4, 8.1 Hz, 1H), 4.01 (tdd, *J* = 12.2, 6.5, 2.5 Hz, 2H), 3.82 (s, 3H), 2.73 (s, 1H), 2.24 (qt, *J* = 12.5, 5.0 Hz, 1H), 1.50 – 1.43 (m, 1H), 1.22 (s, 9H). ^13^C NMR (125 MHz, CDCl_3_) δ 178.81, 159.41, 137.26, 130.35, 128.35, 115.14, 111.88, 100.31, 68.64, 68.52, 67.62, 67.55, 55.48, 38.98, 27.35, 25.76. HR-MS (ESI-TOF) *m/z* for [C_18_H_26_NaO_6_]^+^ ([M+Na]^+^): calculated 361.1627, measured 361.1614.

**5-fluoro-2,4-bis((trimethylsilyl)oxy)pyrimidine [TMS2(F-Uracil)]**

To a solution of 5-F-Uracil (200 mg, 1.53 mmol, 1.0 eq.) in HMDS (1,1,1,3,3,3-hexamethyldisilazane, 10.3 mL, 0.15 M) was added ammonium sulfate (163 mg, 1.23 mmol, 0.8 eq.) at room temperature. The reaction was stirred for 2 h at reflux condition. After reaction finished, mixture was concentrated in vacuo and used for next step without further purification.

**General procedure for MCCB-04-035 (4a), MCCB-04-035 (4b), and their diastereomers (4a′, 4b′)**

Compound **3a/3b** (1.0 eq.) was dissolved in 1% HCl in MeOH (0.15 M) then reaction mixture was stirred for 1 h. After reaction, the mixture was evaporated under pressure, the residue was used for next step without further purification. To a solution of furan pivaloyl intermediate (1.0 eq.) in dichloroethane (DCE, 0.1 M) was added protected nucleobase (1.25 eq.) and tin chloride (SnCl_4_, 1.25 eq.) at –15 ºC. The reaction was stirred for 2 h at 0 ºC. After reaction, sodium bicarbonate was added and stirred 0.5 eq. for quenching. Then mixture was extracted with CH_2_Cl_2_. After concentrated *in vacuo*, the residue purified by flash column chromatography (SiO_2_, 5% MeOH in CH_2_Cl­_2_) to afford nucleobase glycol intermediate. And this intermediate (1.0 eq.) was dissolved in 1.0 M NaOH : 1,4-dioxane = 1:1 (0.2 M) and stirred for 3 h at room temperature. After reaction, mixture was titrated at pH 7 with acetic acid and evaporated. Then diastereomers were purified by HPLC purification (eluent B, 5% to 80% gradient for 30 min, C18 column) to afford compound **4a**/**4b** (13–19% yield, 3 steps) and side **4a′/4b′**.

**5-fluoro-1-((1*R*,3*R*)-3-(hydroxymethyl)-6-methyl-1,3-dihydroisobenzofuran-1-yl)pyram-idine-2,4(1*H*,3*H*)-dione (4a)**

Compound **4a** (58.9 µmol, 19% yield, 3 steps) was synthesized from **3a** (310 µmol) following the described above general procedure. ^1^H NMR (500 MHz, MeOD) δ 7.43 (t, *J* = 2.4 Hz, 1H), 7.34 (t, *J* = 4.3 Hz, 2H), 7.19 (t, *J* = 3.9 Hz, 2H), 5.57 (q, *J* = 3.6 Hz, 1H), 3.90 (dd, *J* = 12.0, 3.5 Hz, 1H), 3.76 (dd, *J* = 12.0, 4.7 Hz, 1H), 2.40 (s, 3H). ^13^C NMR (125 MHz, MeOD) δ 151.39, 140.71, 138.68, 137.76, 132.26, 125.95, 125.68, 123.99, 122.99, 90.10, 86.92, 65.66, 49.62, 49.45, 49.28, 21.27. HR-MS (ESI-TOF) *m/z* for [C_14_H_13_FNaN_2_O_4_]^+^ ([M+Na]^+^): calculated 315.0757, measured 315.0712.

**5-fluoro-1-((1S,3R)-3-(hydroxymethyl)-6-methyl-1,3-dihydroisobenzofuran-1-yl)pyrimidine-2,4(1H,3H)-dione (4a′)**

Compound **4a′** (27.9 µmol, 9% yield, 3 steps) was synthesized from **3a** (310 µmol) following the described above general procedure. ^1^H NMR (500 MHz, MeOD) δ 7.71 (d, 1H), 7.55 (d, *J* = 5.5 Hz, 1H), 7.37 – 7.32 (m, 2H), 7.13 (s, 1H), 5.26 (s, 1H), 4.05 – 3.95 (m, 2H), 2.39 (s, 3H). ^13^C NMR (125 MHz, DMSO) δ 157.13, 156.92, 149.64, 140.63, 138.79, 138.19, 137.25, 136.40, 130.62, 125.31, 125.03, 122.64, 121.86, 86.68, 84.59, 62.28, 20.72. HR-MS (ESI-TOF) *m/z* for [C_14_H_13_FNaN_2_O_4_]^+^ ([M+Na]^+^): calculated 315.0757, measured 315.0766.

**5-fluoro-1-((1*R*,3*R*)-3-(hydroxymethyl)-6-methoxy-1,3-dihydroisobenzofuran-1-yl)pyri-midine-2,4(1*H*,3*H*)-dione (4b)**

Compound **4b** (38.4 µmol, 13% yield, 3 steps) was synthesized from **3b** (296 µmol) following the described above general procedure. ^1^H NMR (500 MHz, MeOD) δ 7.42 (t, *J* = 2.4 Hz, 1H), 7.34 (d, *J* = 8.4 Hz, 1H), 7.19 (d, *J* = 6.2 Hz, 1H), 7.07 (dd, *J* = 8.4, 2.3 Hz, 1H), 6.92 (d, *J* = 2.3 Hz, 1H), 5.52 (dt, *J* = 4.7, 3.3 Hz, 1H), 3.87 (dd, *J* = 12.0, 3.5 Hz, 1H), 3.81 (s, 3H), 3.73 (dd, *J* = 12.0, 4.8 Hz, 1H). ^13^C NMR (125 MHz, MeOD) δ 162.46, 151.41, 143.04, 141.17, 138.90, 133.27, 125.90, 125.63, 124.12, 118.46, 107.90, 90.04, 86.71, 65.75, 56.15, 49.63, 49.46. HR-MS (ESI-TOF) *m/z* for [C_14_H_13_FNaN_2_O_5_]^+^ ([M+Na]^+^): calculated 331.0706, measured 331.0663.

**5-fluoro-1-((1S,3R)-3-(hydroxymethyl)-6-methoxy-1,3-dihydroisobenzofuran-1-yl)pyrimidine-2,4(1H,3H)-dione (4b′)**

Compound **4b′** (23.7 µmol, 8% yield, 3 steps) was synthesized from **3b** (296 µmol) following the described above general procedure. ^1^H NMR (500 MHz, MeOD) δ 7.74 – 7.67 (m, 1H), 7.36 – 7.31 (m, 1H), 7.29 (d, *J* = 8.4 Hz, 1H), 7.06 (dd, *J* = 8.4, 2.3 Hz, 1H), 6.85 (d, *J* = 2.3 Hz, 1H), 5.25 – 5.20 (m, 1H), 3.96 (qd, *J* = 12.5, 2.8 Hz, 2H), 3.79 (s, 3H). ^13^C NMR (125 MHz, DMSO) δ 160.05, 157.26, 157.05, 149.73, 140.69, 138.85, 137.50, 131.99, 125.41, 125.13, 123.03, 116.98, 106.83, 86.65, 84.41, 62.44, 55.51. HR-MS (ESI-TOF) *m/z* for [C_14_H_13_FNaN_2_O_5_]^+^ ([M+Na]^+^): calculated 331.0706, measured 331.0701.

**Spectral Data**

**
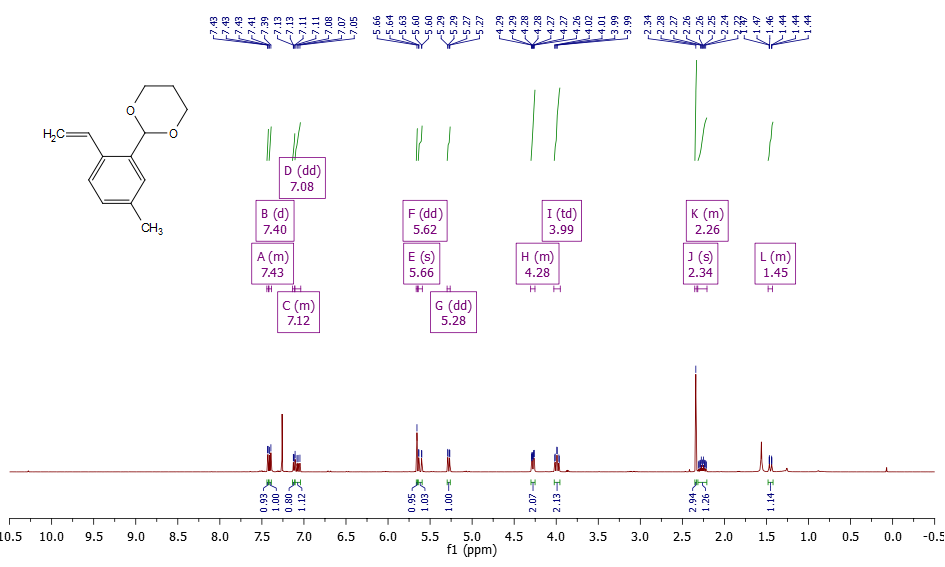
**

^1^H NMR spectrum (500MHz) of compound **2a** in CDCl_3_

**
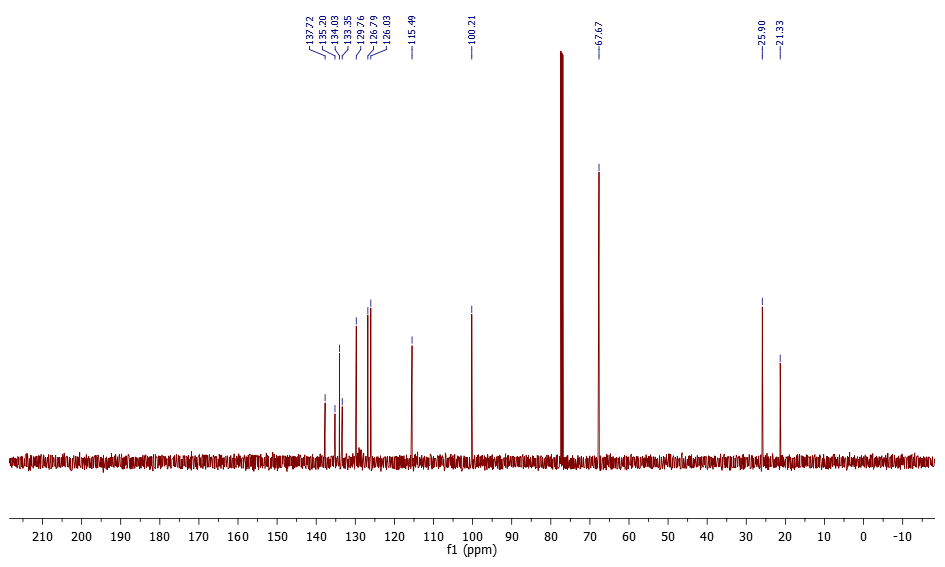
**

^13^C NMR spectrum (125MHz) of compound **2a** in CDCl_3_
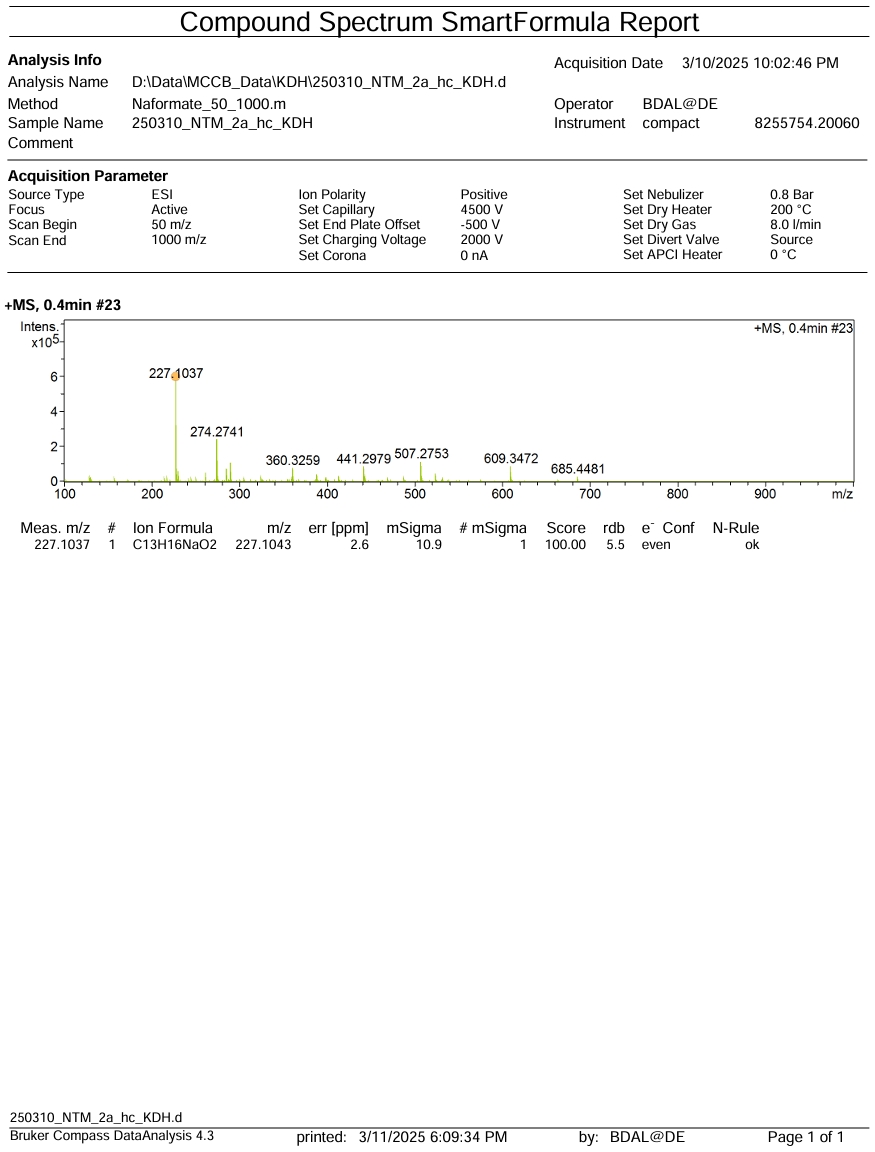


HR-MS data of compound **4a**

HR-MS data of compound **2a**

^
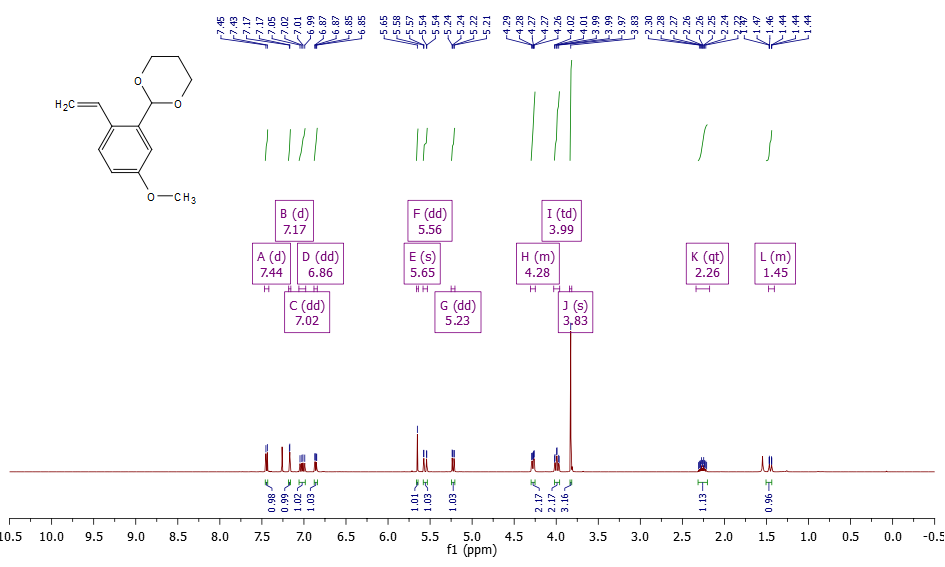
^

^1^H NMR spectrum (500MHz) of compound **2b** in CDCl_3_

**
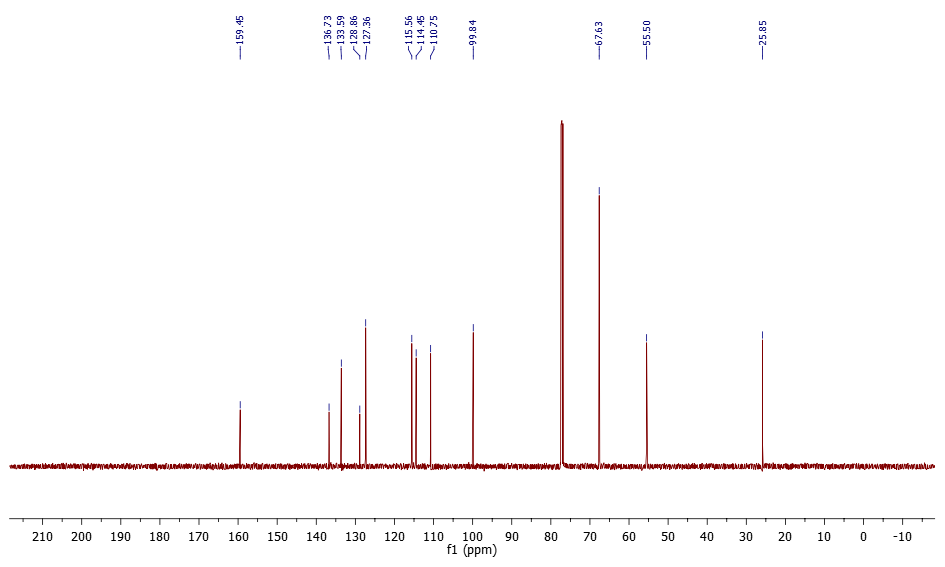
**

^13^C NMR spectrum (125MHz) of compound **2b** in CDCl_3_
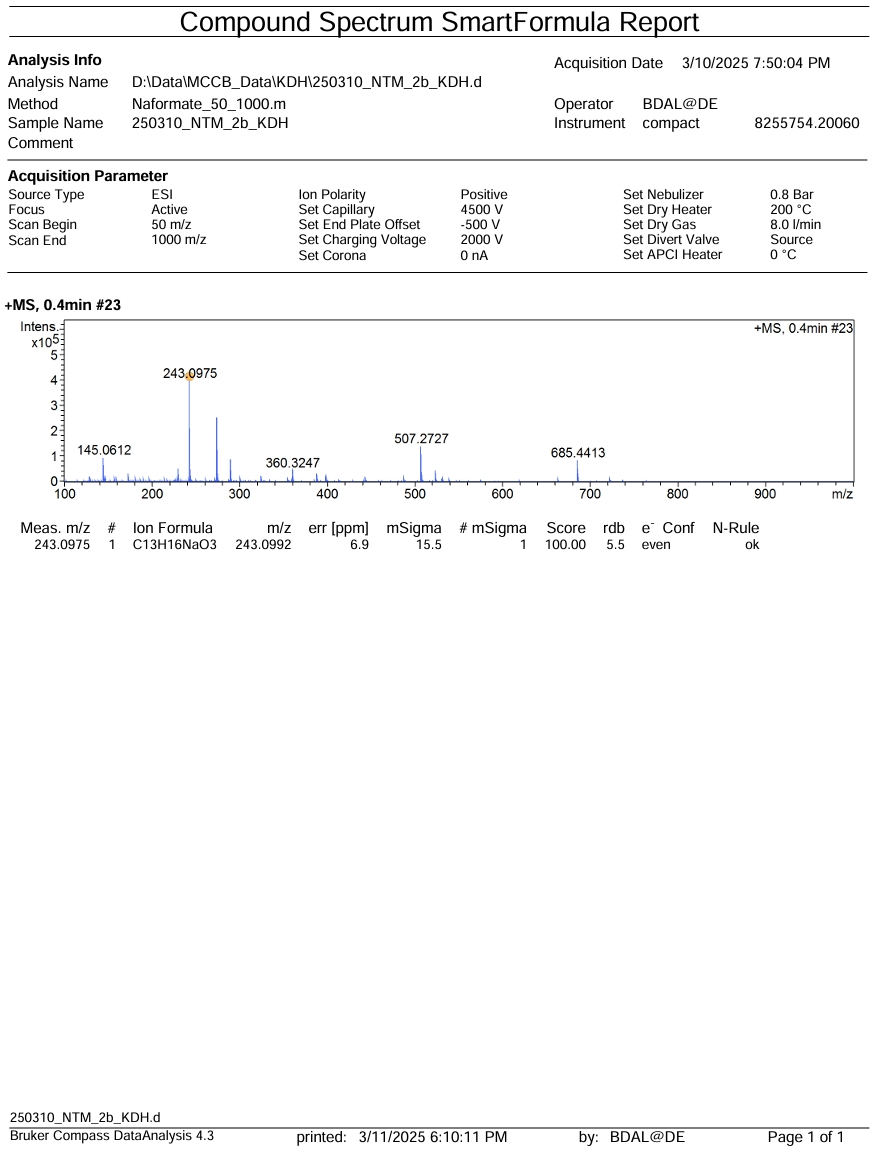


HR-MS data of compound **4a**

HR-MS data of compound **2b**

^
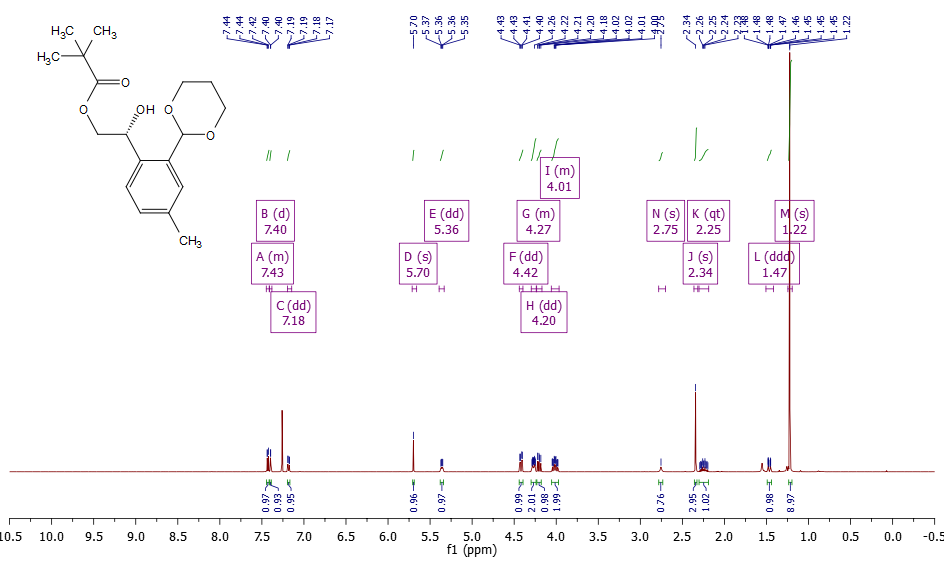
^

^1^H NMR spectrum (500MHz) of compound **3a** in CDCl_3_

^
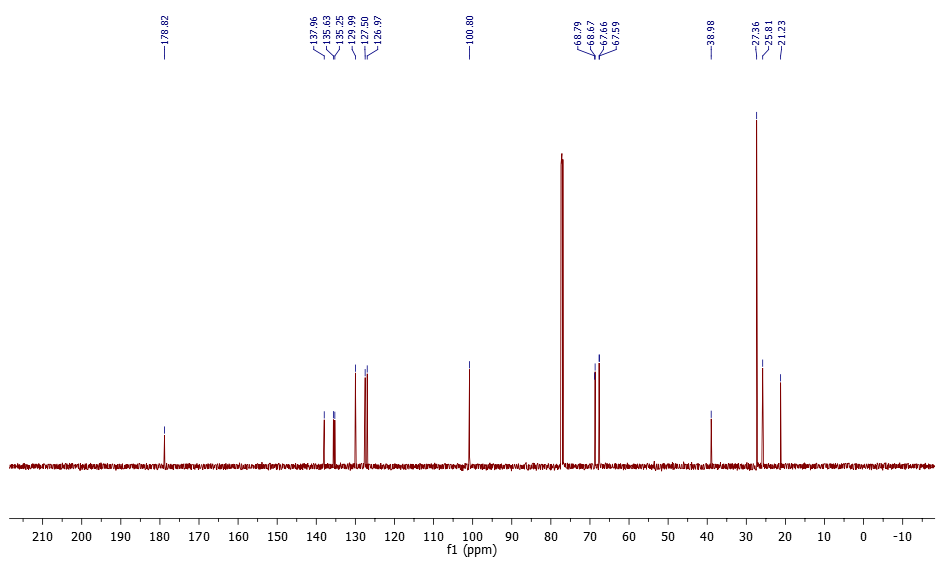
^

^13^C NMR spectrum (125MHz) of compound **3a** in CDCl_3_
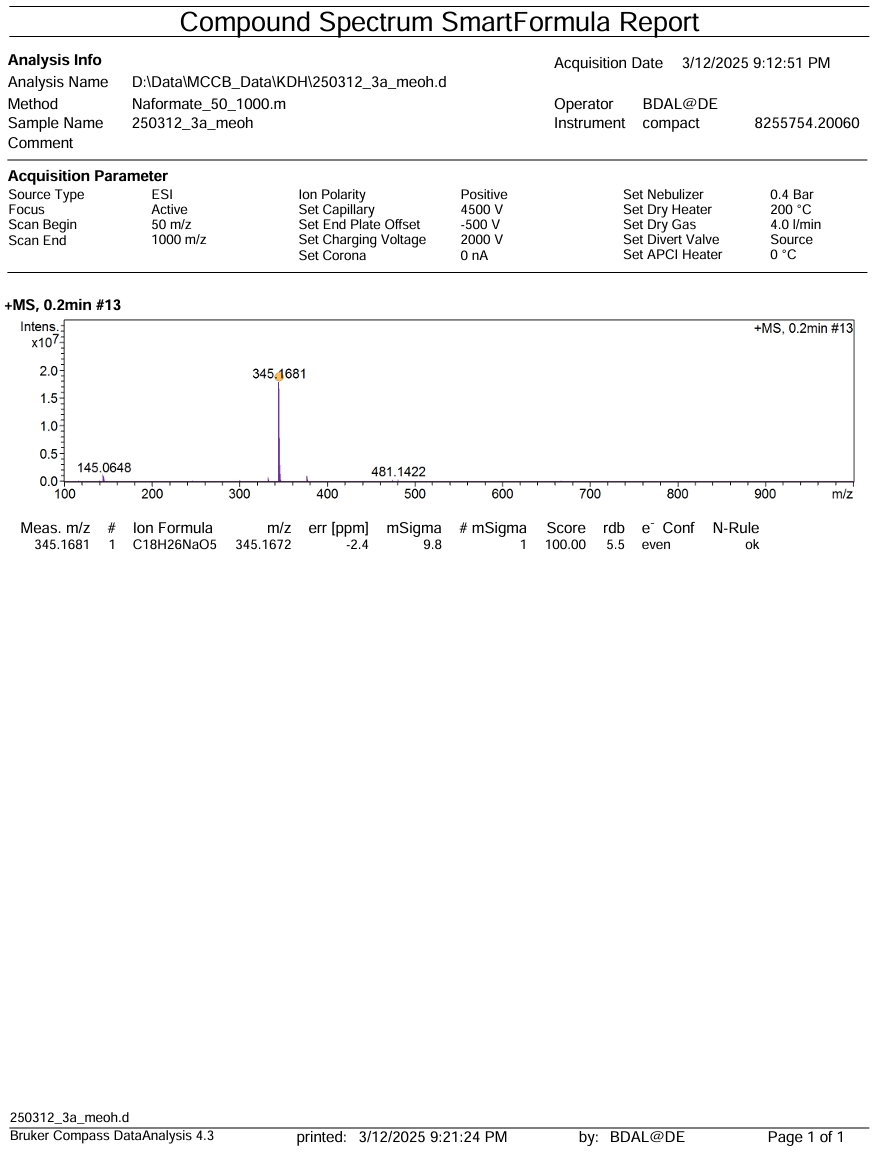


HR-MS data of compound **4a**

HR-MS data of compound **3a**

^
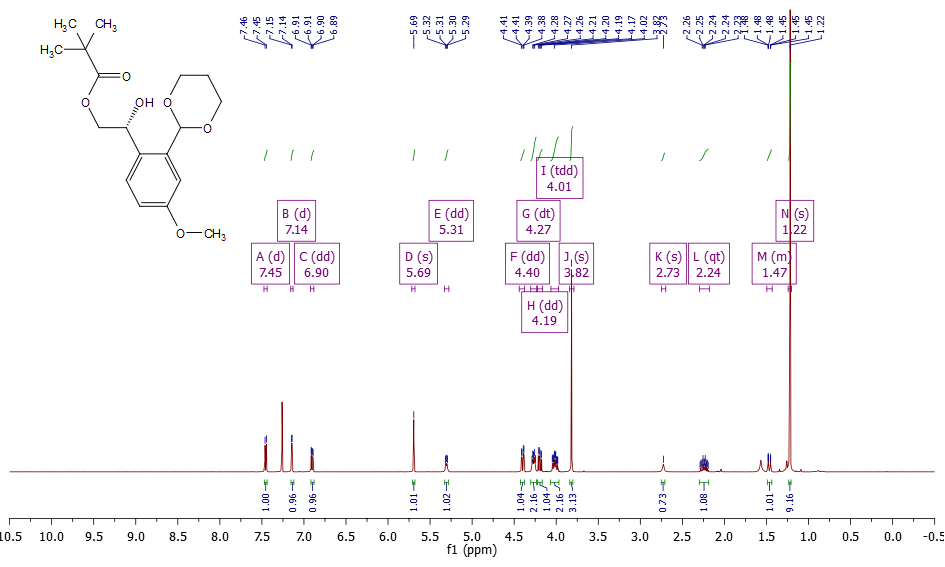
^

^1^H NMR spectrum (500MHz) of compound **3b** in CDCl_3_

**
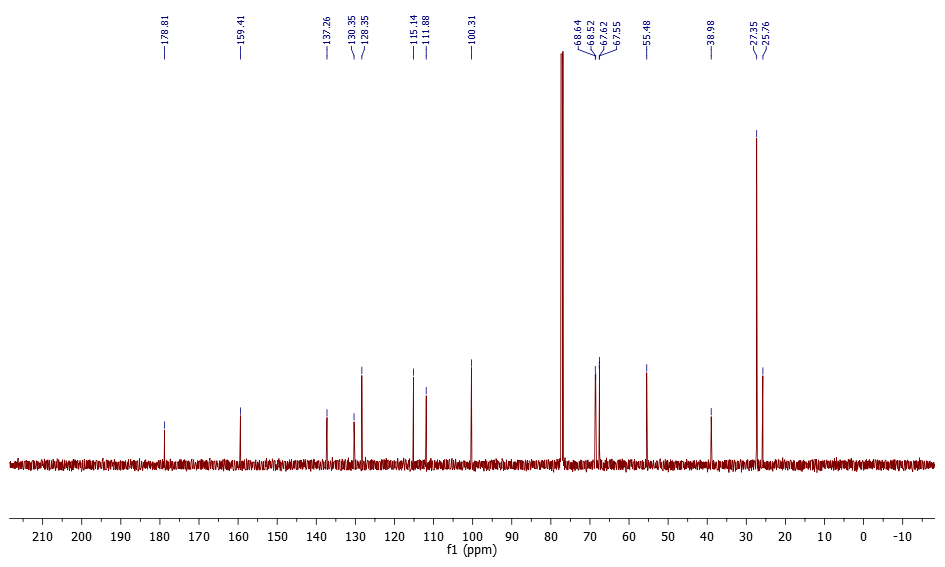
**

^13^C NMR spectrum (125MHz) of compound **3b** in CDCl_3_

HR-MS data of compound **4a**

HR-MS data of compound **4a**.


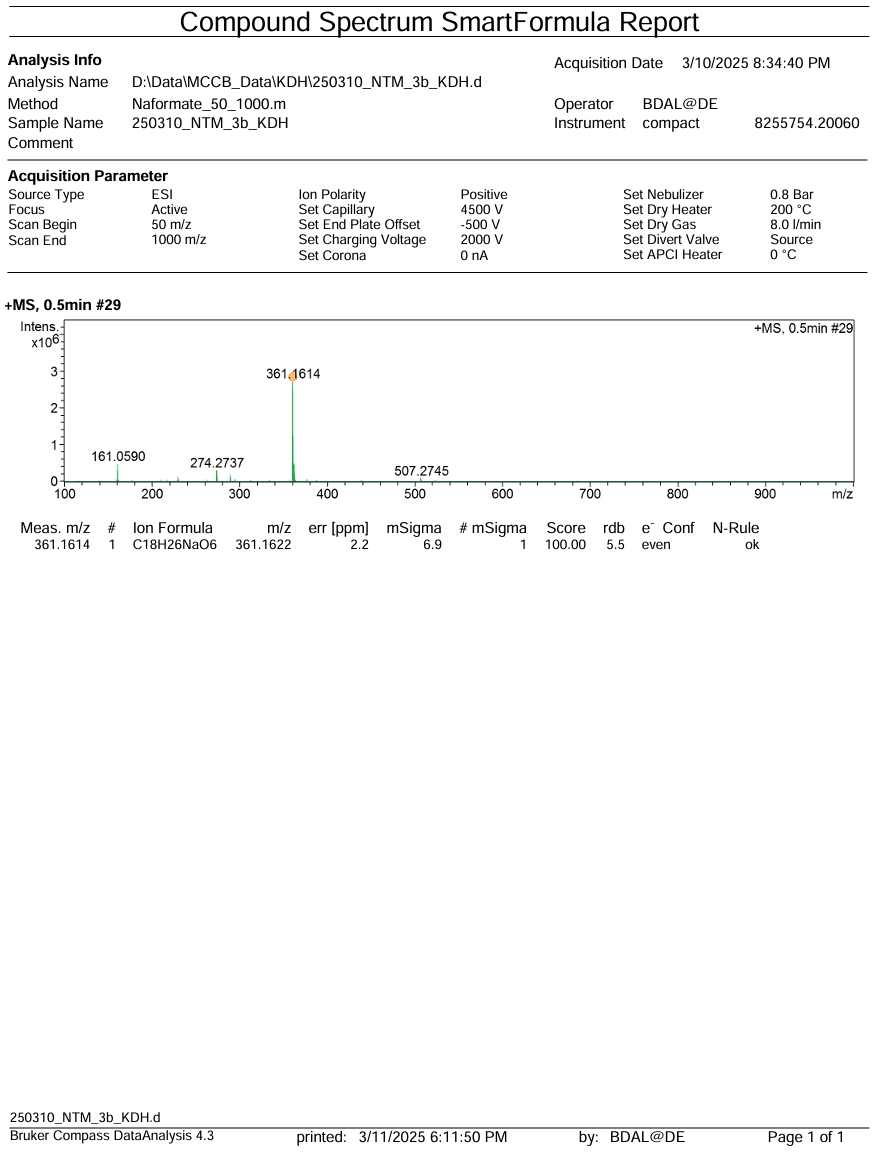


HR-MS data of compound **3b**

^
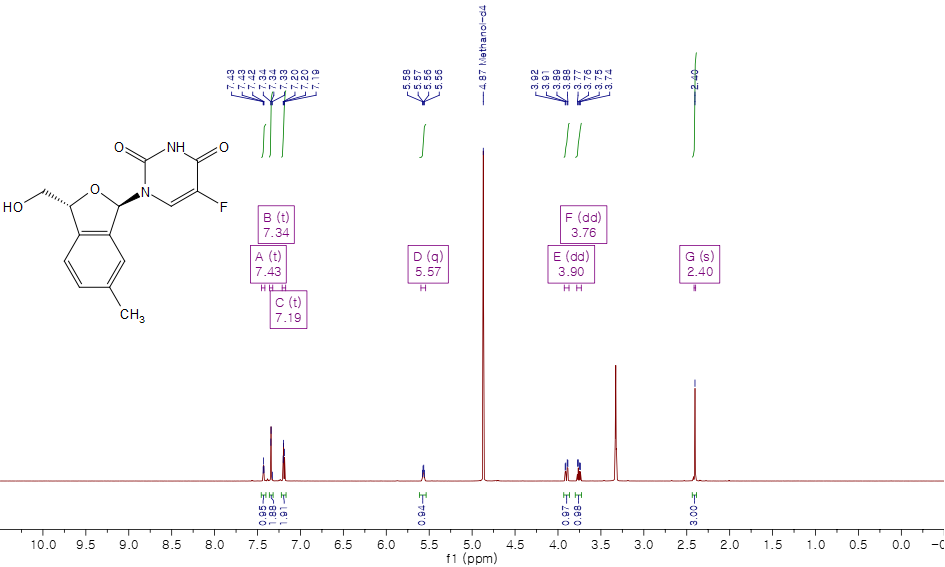
^

^1^H NMR spectrum (500MHz) of compound **4a** in MeOD

**
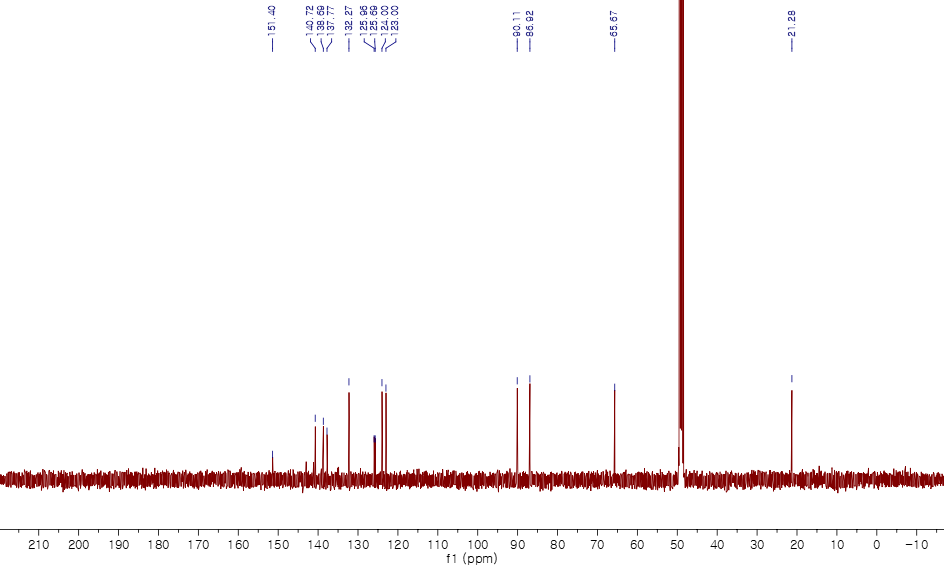
**

^13^C NMR spectrum (125MHz) of compound **4a** in MeOD

HR-MS data of compound **4a**


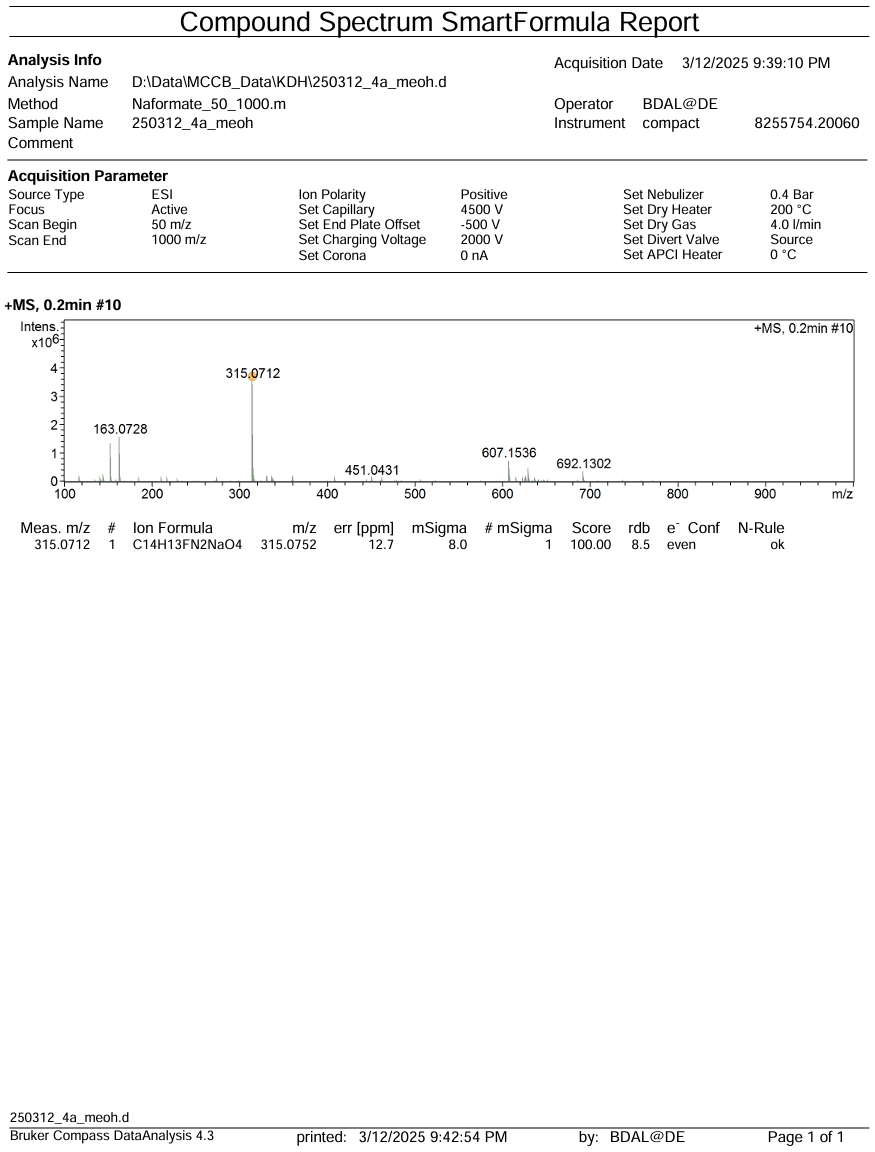


HR-MS data of compound **4a**

**
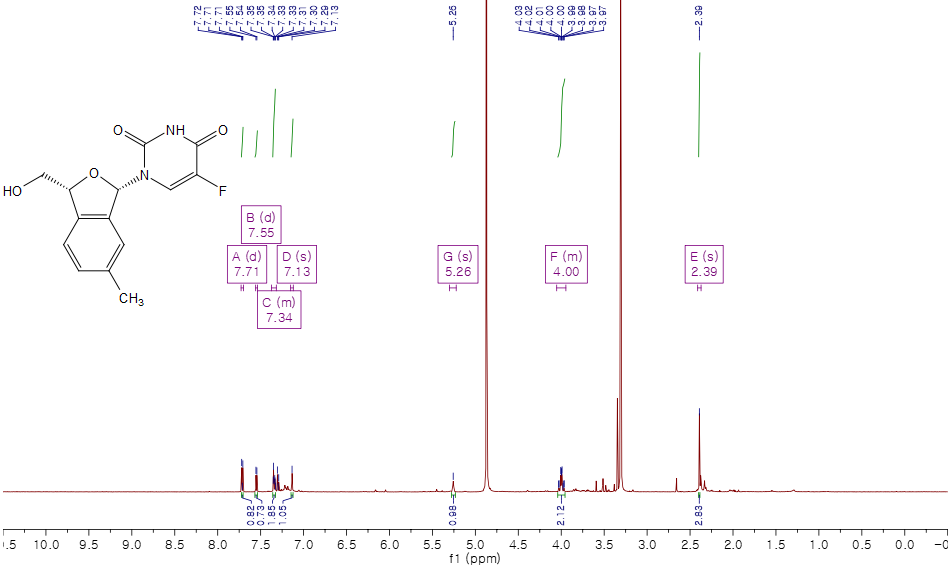
**

^1^H NMR spectrum (500MHz) of compound **4a′** in MeOD

**
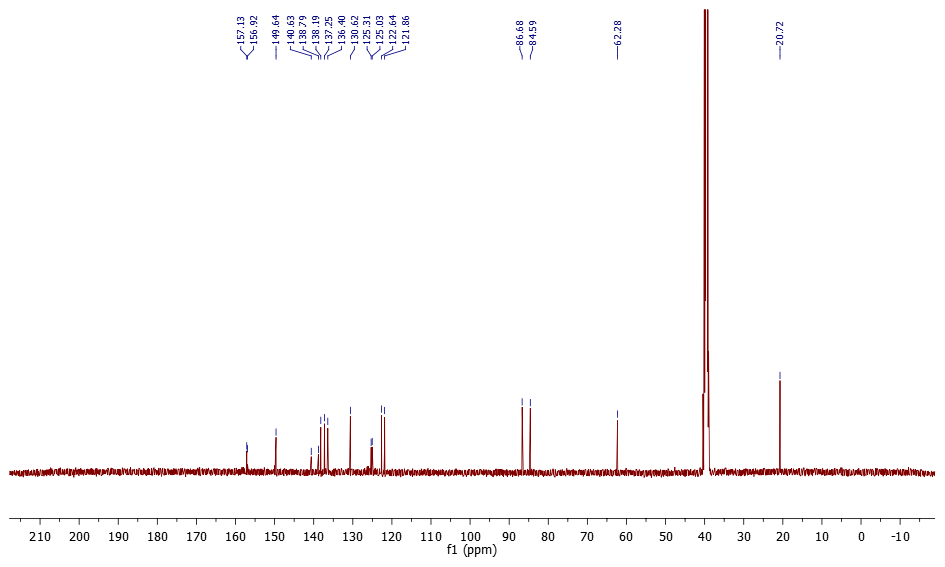
**^13^C NMR spectrum (125MHz) of compound **4a′** in DMSO

HR-MS data of compound **4a**


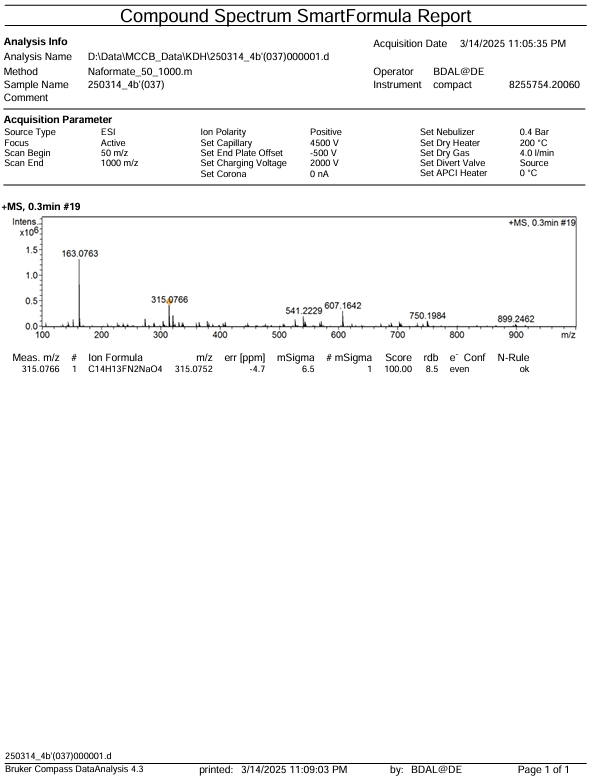
HR-MS data of compound **4a′**

^1^H NMR spectrum (500MHz) of compound **4b** in MeOD

**
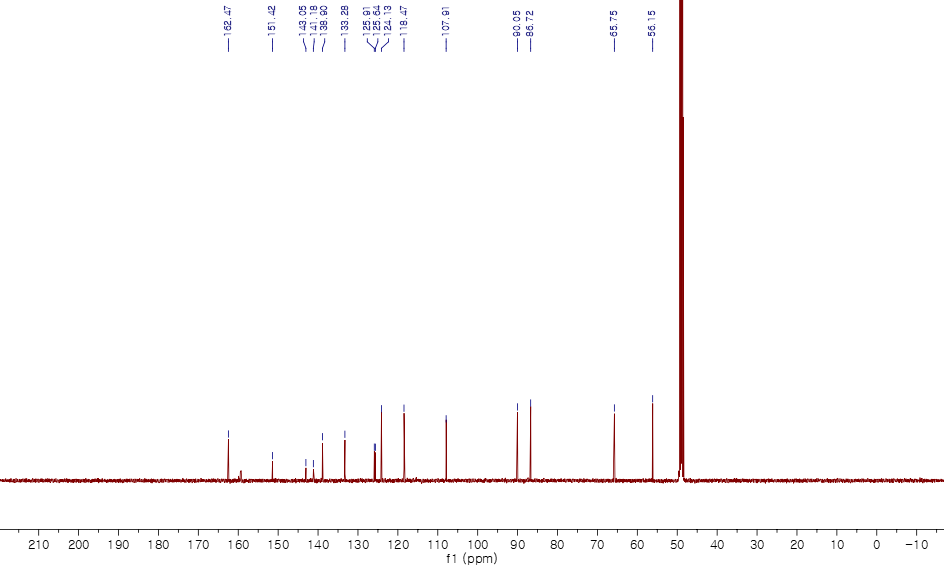
**

^13^C NMR spectrum (125MHz) of compound **4b** in MeOD
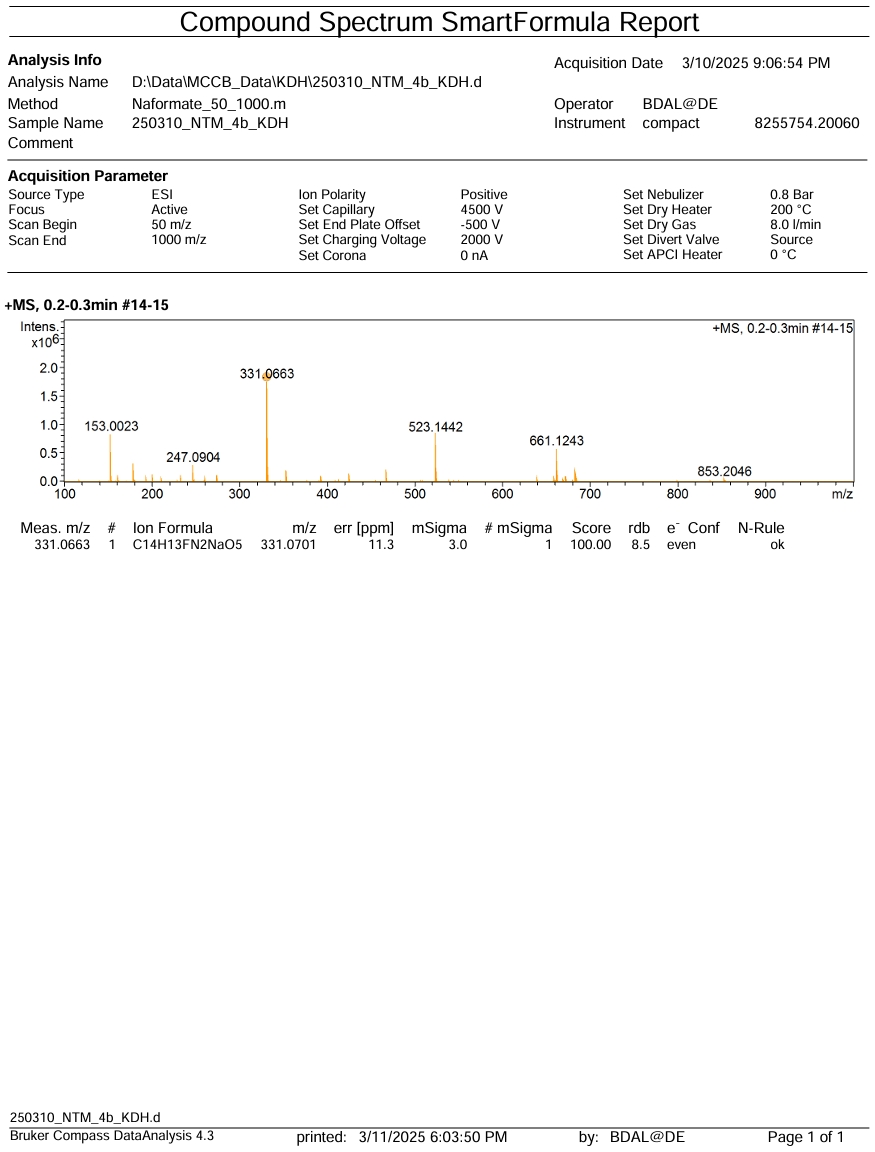


HR-MS data of compound **4b**

**
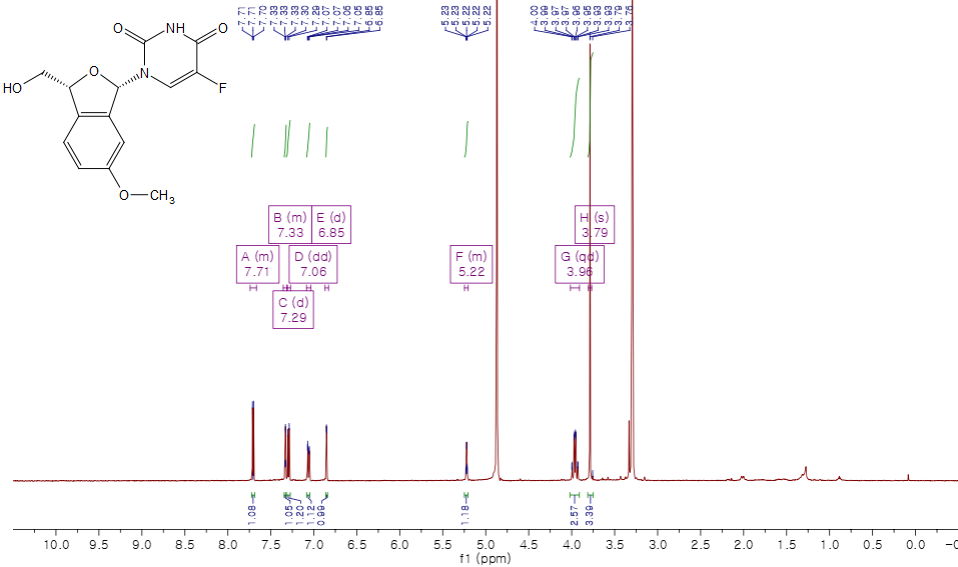
**

^1^H NMR spectrum (500MHz) of compound **4b′** in MeOD

**
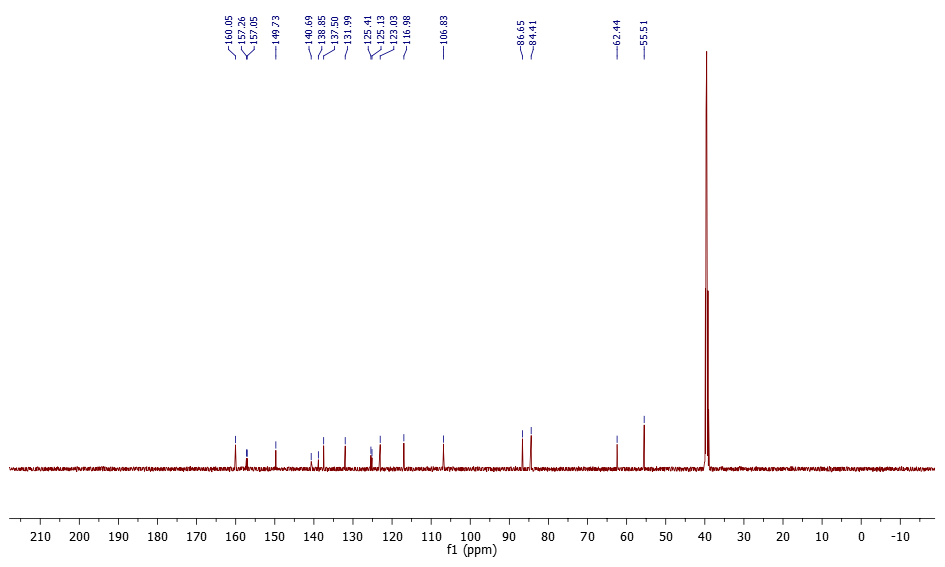
**

^13^C NMR spectrum (125MHz) of compound **4b′** in DMSO

HR-MS data of compound **4a**


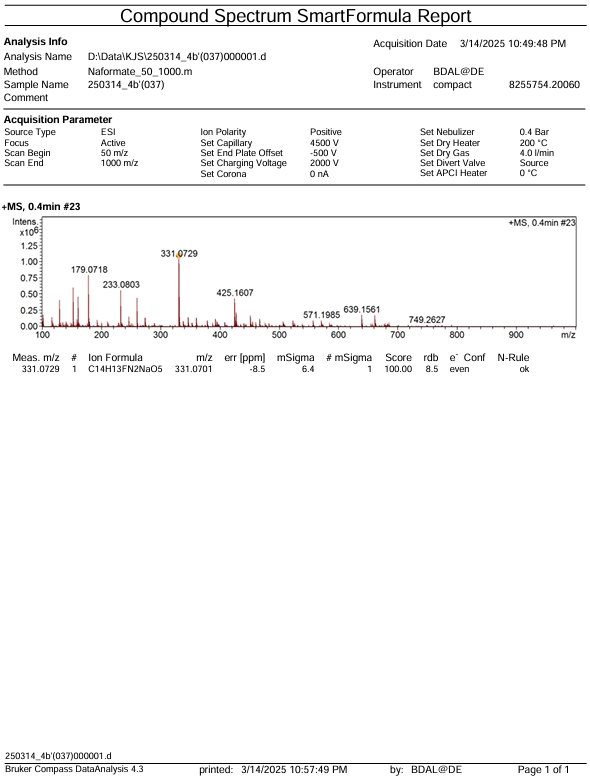
HR-MS data of compound **4b′**
